# Transgenic and physiological mouse models give insights into different aspects of amyotrophic lateral sclerosis

**DOI:** 10.1242/dmm.037424

**Published:** 2019-01-02

**Authors:** Francesca De Giorgio, Cheryl Maduro, Elizabeth M. C. Fisher, Abraham Acevedo-Arozena

**Affiliations:** 1 Department of Neuromuscular Diseases, UCL Institute of Neurology, and MRC Centre for Neuromuscular Disease, University College London, Queen Square, London WC1N 3BG, UK; 2 Unidad de Investigación Hospital Universitario de Canarias, Fundación Canaria de Investigación Sanitaria and Instituto de Tecnologías Biomédicas (ITB), La Laguna, 38320 Tenerife, Spain

**Keywords:** Amyotrophic lateral sclerosis, ALS, Transgenic, Knock-in, ENU, Gene targeted

## Abstract

A wide range of genetic mouse models is available to help researchers dissect human disease mechanisms. Each type of model has its own distinctive characteristics arising from the nature of the introduced mutation, as well as from the specific changes to the gene of interest. Here, we review the current range of mouse models with mutations in genes causative for the human neurodegenerative disease amyotrophic lateral sclerosis. We focus on the two main types of available mutants: transgenic mice and those that express mutant genes at physiological levels from gene targeting or from chemical mutagenesis. We compare the phenotypes for genes in which the two classes of model exist, to illustrate what they can teach us about different aspects of the disease, noting that informative models may not necessarily mimic the full trajectory of the human condition. Transgenic models can greatly overexpress mutant or wild-type proteins, giving us insight into protein deposition mechanisms, whereas models expressing mutant genes at physiological levels may develop slowly progressing phenotypes but illustrate early-stage disease processes. Although no mouse models fully recapitulate the human condition, almost all help researchers to understand normal and abnormal biological processes, providing that the individual characteristics of each model type, and how these may affect the interpretation of the data generated from each model, are considered and appreciated.

## Abstract (precis)

**Summary:** This Review compares two key types of amyotrophic lateral sclerosis (ALS) mouse models: transgenic mice and those that express genes at physiological levels. We explore the advantages of each type for studying pathomechanisms, to understand what they can teach us about ALS.

## Introduction

Amyotrophic lateral sclerosis (ALS) is a progressive neurodegenerative disorder first described in 1869 by Jean-Martin Charcot (Charcot and Joffroy, 1869). It has a mean incidence of ∼2/100,000 worldwide and a prevalence of ∼6/100,000 in Europe (Costa and de Carvalho, 2016; Marin et al., 2016), with a lifetime risk of ∼1 in 300 in Western populations (Brown and Al-Chalabi, 2017). ALS patients typically present a focal onset, starting as unilateral limb weakness or bulbar impairment. Clinical symptoms usually start in mid-life and are a consequence of the dysfunction and death of motor neurons (MNs) in the primary motor cortex, brainstem and spinal cord, which causes spasticity, weakness and muscle wasting, gradually leading to paralysis and death from respiratory failure, typically less than 5 years from diagnosis (Huynh et al., 2016; Van Damme et al., 2017).

There are no effective treatments for ALS apart from daily care and support to counteract the symptoms. Currently, there are only two US Food and Drug Administration (FDA)- and European Medicines Agency (EMA)-approved neuroprotective drugs that increase the lifespan of some patients by a few months: Riluzole, which blocks excessive glutamatergic neurotransmission, and Edaravone, which prevents oxidative stress damage.

Although 90% of ALS patients have sporadic (sALS) disease without apparent family history, ∼5-10% of cases are familial (fALS), usually showing monogenic autosomal dominant inheritance (Brown and Al-Chalabi, 2017). In 1993, the first causative gene for ALS was discovered, encoding the enzyme Cu/Zn superoxide dismutase 1 (*SOD1*) (Rosen et al., 1993). Research shows that *SOD1*-ALS accounts for ∼20% of fALS and ∼2% of sALS, with >150 mutations identified throughout the coding region and causing an unknown toxic gain of function (GOF) (Saccon et al., 2013; Kaur et al., 2016). SOD1 is ubiquitously expressed and important for the removal of free radicals, although it likely has other non-canonical roles; for example, as a transcriptional regulator under oxidative stress, possibly as an RNA-binding protein and a signalling molecule (Bunton-Stasyshyn et al., 2015).

Since the discovery of *SOD1*’s association with ALS, mutations in more than 20 genes were found to be causative, most with an autosomal-dominant pattern of transmission, together with >30 potential disease-modifying genes (Li and Wu, 2016). Causative genes include the chromosome 9 open reading frame 72 (*C9ORF72*), in which an intronic hexanucleotide repeat expansion gives rise to ALS. This mutation is the most common cause of fALS, and is found in up to 40% of fALS and ∼9% of sALS in Caucasians (DeJesus-Hernandez et al., 2011; Renton et al., 2011; Goldstein et al., 2018). Other well known ‘ALS genes’ include TAR DNA-binding protein (*TARDBP*; encoding TDP-43), found in ∼5% of fALS and ∼2% of sALS, and fused in sarcoma (also known as FUS RNA-binding protein; *FUS*), found in ∼6% of fALS and ∼1% of sALS (Ingre et al., 2015; Tarlarini et al., 2015). TDP-43 and FUS are RNA-binding heterogeneous nuclear ribonucleoproteins (hnRNPs) mainly localised in the nucleus, and are involved in mRNA splicing, gene transcription and microRNA maturation, mRNA shuttling from the nucleus to the cytoplasm and stress granule formation. Cytoplasmic mislocalisation and nuclear depletion of TDP-43 is a key feature of most ALS cases and may contribute to disease pathogenesis (Guerrero et al., 2016). Protein aggregates containing truncated hyperphosphorylated and/or ubiquitinated TDP-43 are found within MNs in >95% of ALS-affected brains and spinal cords (Chou et al., 2018), and can occur in other neurological disorders, including Alzheimer’s, Parkinson’s and Huntington’s diseases, highlighting the importance of TDP-43 in neurodegeneration (Liu et al., 2017; St-Amour et al., 2018).

Other genes less frequently mutated in ALS include coiled-coil-helix-coiled-coil-helix domain-containing 10 (*CHCHD10*) (Bannwarth et al., 2014), kinesin family member 5A (*KIF5A*) (Brenner et al., 2018), matrin 3 (*MATR3*) (Johnson et al., 2014), optineurin (*OPTN*) (Maruyama et al., 2010), profilin 1 (*PFN1*) (Wu et al., 2012)*,* senataxin (*SETX*) (Chen et al., 2004), sequestosome 1 (*SQSTM1/p62*) (Fecto, 2011), TANK-binding kinase 1 (*TBK1*) (Cirulli et al., 2015; Freischmidt et al., 2015), ubiquilin 2 (*UBQLN2*) (Deng et al., 2011), valosin-containing protein (*VCP*) (Johnson et al., 2010) and VAMP-associated protein B and C (*VAPB*) (Nishimura et al., 2004). As each new gene is identified, the next step is to make a mouse model. There are different types of mutant mice, which yield different insights and should be used to address different research questions.

## Mouse models of ALS

**Fig. 1.**
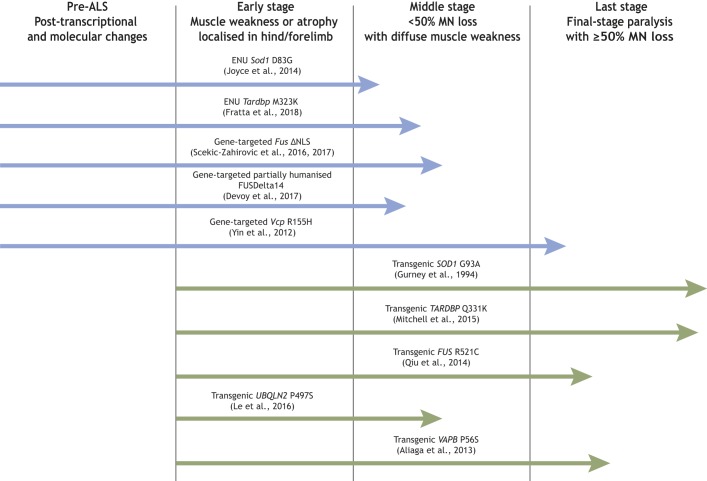
**Features of transgenic versus physiological mouse models for studying ALS.** Examples from Table 1, showing potential windows of ALS pathology to investigate using transgenic or physiological mouse models; lengths of arrows correspond, approximately, to the severity of the phenotype on either heterozygous or homozygous mice at the oldest age measured, as per the references. We note that with respect to ALS genetic models, the *SOD1* G93A (Gurney et al., 1994) mouse was the first transgenic line. We believe that *Vcp* R155H (Badadani et al., 2010; Yin et al., 2012) was the first gene-targeted model, the *Sod1* D83G (Joyce et al., 2015) line was the first ENU mouse model, and the FUSDelta14 model (Devoy et al., 2017) was the first genomically humanised knock-in to the endogenous mouse locus, although this is a partial humanisation; see Table 1.

We know little of early-stage ALS pathomechanisms, and we still have a lot to learn about the disease trajectories for fALS and sALS. Here, we discuss the main features of the different types of mouse models that are helping us to elucidate the molecular pathology of ALS and its phenotypic implications: transgenic mice, and targeted and ENU mutant mice (Fig. 1). We then focus on comparing the phenotypes of mice with ALS gene mutations for which at least two of these types of model have been published; namely, *FUS*, *SOD1*, *TARDBP*, *VAPB*, *VCP* and *UBQLN2*.

**Table 1.**
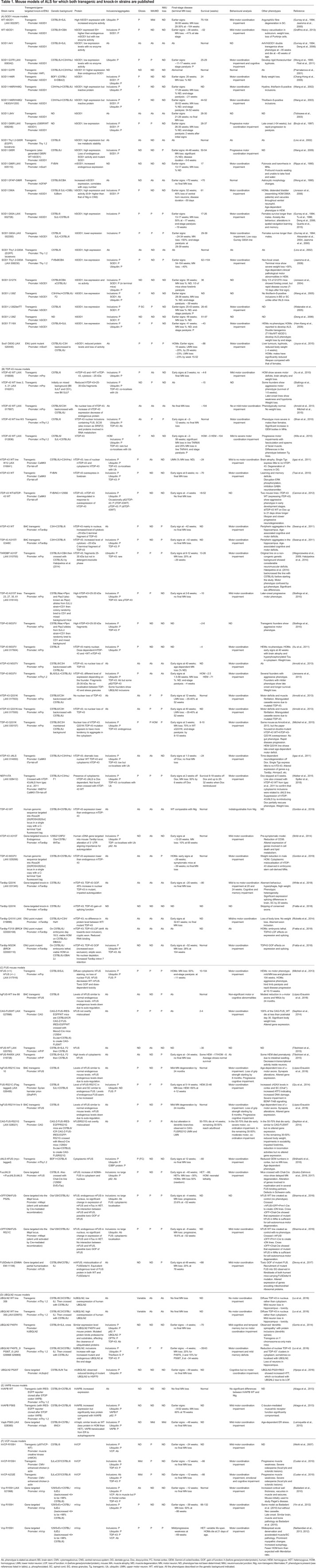
**Mouse models of ALS for which both transgenic and knock-in strains are published**

## Transgenic mouse models

ALS is mostly an autosomal-dominant disorder and therefore the majority of mouse models have been transgenic lines, made by randomly inserting human (in most cases) mutant ALS genes into the mouse genome (Table 1). This is a fast method of producing new strains and, because the disease is dominant, the phenotype usually manifests, despite the presence of intact orthologous mouse genes. Indeed, the first model of ALS, the *SOD1^G93A^* transgenic strain [Tg(SOD1*G93A)1Gur], was published a year after the discovery of *SOD1*-ALS mutations in humans (Gurney et al., 1994) (Table 1A) and remains the most commonly used ALS mouse model. Owing to the early onset, fast disease progression towards an early humane endpoint, progressive MN loss and low variability of the phenotype on defined genetic backgrounds, the *SOD1^G93A^* transgenic strain has become the workhorse for testing therapeutics aimed at ameliorating ALS.

Around 30 *FUS* and *TARDBP* mutant transgenic lines have also been created, with variable levels of MN degeneration (Table 1B,C). In contrast, only one of the published *UBQLN2* transgenic lines, carrying the P497S mutation, which disturbs proteasomal degradation, shows motor impairment, mild MN loss (20%) and cytoplasmic aggregates positive for ubiquitin and TDP-43 (Le et al., 2016) (Table 1D). The only mouse model expressing a mutated VAPB protein has a progressive phenotype resulting in ∼60% MN loss by 78 weeks of age (Aliaga et al., 2013) (Table 1E), whereas results are mixed for the VCP transgenic models (Table 1F). Despite the variability in phenotype presentation, transgenic mice remain a critical resource for understanding neurodegeneration but, like all mouse models, they have generic characteristics we need to take into account, as discussed below.

### Site of insertion

Transgene DNA is usually microinjected into fertilised eggs and randomly inserts into the host mouse genome. This can lead to insertional mutagenesis from disrupting a host gene, producing an aberrant phenotype, which is why multiple founder lines from independent transgenic embryos are studied – to be confident that the common phenotypes arise from the transgene. Almost all transgenic lines in Table 1 do not have information on the insertion site, as is the case for the vast majority of transgenic models of neurodegenerative disease (Tosh et al., 2017; Goodwin et al., 2017). Fortunately, in *SOD1^G93A^* mice, the transgene insertion site does not disrupt a known gene (Srivastava et al., 2014; Achilli et al., 2005).

### Transgene copy number and gene expression

Transgenic DNA tends to concatemerise as it inserts into the genome, leading to multiple copies of the exogenous sequence. This results in the overexpression of the protein of interest, often leading to accelerated phenotypes. Furthermore, a caveat to studying transgenic mice arises from the development of aberrant phenotypes due to overexpression. The *SOD1^G93A^* model used most commonly carries ∼25 copies of the human transgene, resulting in overexpression of the protein (Gurney et al., 1994; Shibata, 2001), with MN degeneration progressing rapidly: disease onset occurs at ∼90 days and the humane endpoint occurs by ∼130 days of age, depending on the genetic background of the mouse. However, transgenic mice expressing wild-type human SOD1 at a similar level to mice expressing the mutant transgene have neurological phenotypes likely arising from overexpression and not from mutation, including spinal cord vacuolation with early signs of paresis in one or more limbs (Jaarsma et al., 2000) and even MN loss (Graffmo et al., 2013). Thus, the ideal controls for mutant transgenic mice are transgenic animals expressing the wild-type transgene at similar levels to the mutant mice to control for the effects of overexpression per se. However, the wild-type human SOD1 transgenic lines are not without problems. For example, transgene insertion sites have not been assessed, and although they develop phenotypes relevant to MN disease, these are more profound in some of the mutant SOD1 transgenic lines, such as the *SOD1^G93A^* model. Nevertheless, a large proportion of ALS studies in mutant transgenic mice do not use wild-type transgenic controls, and this is an option that should at least be considered for future work.

Some genes are highly dosage sensitive and a subtle deviation from the physiological levels leads to aberrant phenotypes, even when the protein product is wild type. Many of the RNA-binding proteins that cause ALS when mutated belong to this category, including TDP-43 and FUS (Table 1B,C). For example, transgenic mice overexpressing wild-type human *TARDBP* (from a Thy1.2 promoter) by 1.2× to 2× fold over the endogenous gene level have 25% MN loss with rare cytoplasmic inclusions containing TDP-43 (Wils et al., 2010). Overexpression of human wild-type *FUS* (under the mouse prion promoter) results in aggregation of human FUS protein and 60% loss of MNs in homozygous transgenic mice, leading to a more severe phenotype in homozygotes than in hemizygotes (Mitchell et al., 2013) (Table 1C). Indeed, RNA-binding proteins such as TDP-43 often control the expression levels of their own transcript through autoregulation. Therefore, when transgene expression levels of wild-type or mutant proteins rise above a threshold, the expression levels of the mouse endogenous transcripts are reduced, possibly contributing towards toxicity.

Furthermore, transgenes are often engineered to have exogenous promotors to ensure high levels of expression in the tissues of interest, but such ectopic expression can result in novel phenotypes. For example, two unrelated transgenic mouse lines overexpressing VCP with the R155H mutation, under the control of a muscle creatine kinase (mMCK) or a cytomegalovirus (CMV) promotor, have differences in the survival and presence of cytoplasmic aggregates containing VCP, and variability in the levels of motor impairment (Table 1F) (Weihl et al., 2007; Custer et al., 2010). Similarly, transgenic mice overexpressing mutant human *TARDBP^A315T^* driven by the mouse prion promoter (the activity of which is strong in neurons, although it is also widely expressed in other cell types) unexpectedly die early from neurodegeneration in the gut rather than in MNs (Wegorzewska et al., 2009; Hatzipetros et al., 2014).

Finally, the transgene array may alter copy number at meiosis; thus, colonies need to be monitored constantly because the transgene’s copy number usually determines phenotype severity. For example, the Tg(SOD1*G93Adl)1Gur (*SOD1G93Adl*; also known as G1del) mice appear to have arisen from a deletion in the transgene array of a *SOD1^G93A^* mouse (http://jaxmice.jax.org/strain/002300.html). The resulting ‘low copy’ *SOD1^G93A^* transgenic mouse strain carries ∼8-10 copies of the human *SOD1G93A* transgene instead of the ∼25 in the progenitor line, and these ‘low copy’ animals develop paralysis between 24 and 34 weeks of age, considerably later than the ‘high copy’ progenitor line (Alexander et al., 2004; Acevedo-Arozena et al., 2011).

### BAC transgenic mice

Most – but not all transgenic animals – have been made with the longest known complementary DNA (cDNA) sequence for the gene of interest; this is usually because of constraints on DNA insert size in the plasmid vectors used to subclone the transgenic constructs. To avoid this size limit and to generate mice carrying the full genomic architecture of a gene (which is particularly important in the case of *C9ORF72*-ALS, for which the mutation is intronic), researchers can generate transgenic mice with bacterial artificial chromosome (BAC) vectors, which can carry inserts of up to ∼200 kb. This approach was used to generate, for example, C9ORF72 (Balendra and Isaacs, 2018), TDP-43 (Swarup et al., 2011) and FUS (López-Erauskin et al., 2018) BAC transgenic mice. BACs randomly insert into the mouse genome, but generally with very low copy numbers (one to three copies), limiting the effects of overexpression of the gene of interest, although even subtle overexpression can alter the phenotype. As with all transgenics, there is the undesired possibility of insertion mutagenesis, in which integration of the transgene can disrupt an important gene.

### Generic transgenic mouse features for ALS research

Until recently, transgenics were the fastest technology to obtain genetically modified mice, but this is changing as CRISPR/Cas9-based technologies develop. As discussed above, phenotypes can be rapid and severe in transgenic models because of expression of the transgene above endogenous levels. This is helpful for understanding the advanced stages of disease, which in the natural history of ALS is comparable to when most patients receive the diagnosis. Several transgenic models can have quantifiable, progressive loss of MNs severe enough to lead to profound locomotion defects and paralysis during the mouse lifespan (Table 1). These features made them the models of choice for pre-clinical studies and, until recently, almost all ALS therapeutics were solely tested on SOD1 transgenic models. This provides some explanation for the past failures of translating promising therapeutics from SOD1 transgenics to ALS patients, 98% of whom do not suffer from *SOD1*-ALS (Urushitani et al., 2007; Turner and Talbot, 2008; Riboldi et al., 2011; Vallarola et al., 2018).

## Mouse models with mutations at physiological levels in endogenous genes

### Gene-targeted and ENU mutant strains

Mouse models of ALS can be generated by mutating mouse gene orthologues, to express the relevant protein at physiological levels. Here, we discuss the two key types of model with mutations in endogenous genes, produced from gene-targeting strategies or by random mutagenesis with the chemical *N*-ethyl-*N*-nitrosourea (ENU). We describe both as ‘physiological’ models in this article, as ‘knock-in’ (KI) is generally used for gene-targeted mice because it implies purposely engineering the mouse genome.

### Gene-targeted models of ALS

Gene targeting entails introducing specific changes to a DNA sequence of interest. In mice, perhaps its most common use has been to create knockout (KO) animals in which the gene no longer functions, usually to help us understand the biology of individual genes. For example, the International Mouse Knockout program aims to functionally KO each mouse gene, providing phenotypic data for each KO line under the International Mouse Phenotyping program (Muñoz-Fuentes et al., 2018).

#### Gene KOs

Although most forms of ALS appear to be caused by toxic dominant GOF, KO models are an important resource as they can reveal not only critical gene function but also whether there is a loss-of-function (LOF) component to disease pathogenesis. For example, TDP-43 is usually depleted from the nucleus of MNs in TDP-43-ALS, presumably leading to a loss of nuclear TDP-43 function. Although homozygous TDP-43 KO mice are not viable, and heterozygous KO mice express a normal amount of TDP-43 protein due to its autoregulation, conditional TDP-43 KO lines and a transgenic line expressing small interfering RNA against TDP-43 develop MN degeneration (Kraemer et al., 2010), showing that acute TDP-43 LOF can be a driver of neurodegeneration. In *SOD1*-ALS, LOF can play a role in disease pathogenesis, as *Sod1* KO mice develop a severe peripheral neuropathy, leading to denervation (Fischer et al., 2011) and *SOD1*-ALS patients generally have diminished SOD1 dismutase activity (Saccon et al., 2013).

#### KI mutations

Gene targeting has been used to insert specific mutations, usually (but not always; see Sharma et al., 2016; Gordon et al., 2019) into the endogenous mouse gene, with the aim of maintaining physiological expression levels of the (mutant) protein. This approach has been used thus far for *Fus*, *Tardbp*, *Vapb*, *Vcp* and *Ubqln2* mutations*.*

Classical gene targeting involves creating recombinant vectors for homologous recombination in mouse embryonic stem cells, which can be time-consuming and relatively expensive. However, CRISPR/Cas9 targeting in zygotes has made the production of gene-targeted mice – for example, such as two recently described strains recapitulating the human *TARDBP* Q331K mutation (Fratta et al., 2018; White et al., 2018) – considerably more efficient, faster and cheaper. Nevertheless, the possible off-target effects of this technology must be taken into account (Zhang et al., 2015).

One of the first KIs to model ALS was the *Vcp*^R155H^ strain (Badadani et al., 2010; Yin et al., 2012). These mice have age-dependent degeneration of ventral horn MNs with up to 50% MN loss, TDP-43-positive cytoplasmic inclusions, mitochondrial aggregation and progressive astrogliosis. These and other *Vcp* KI mice do not have rapidly progressive fatal ALS features, but they are important for understanding the onset of ALS (Table 1F).

Site-directed insertion of exogenous DNA into known ‘safe harbour’ sites in the genome, such as the *Rosa26* locus, also uses homologous recombination and is an alternative that avoids the random insertion mutagenesis of transgenic approaches. For example, TDP-43 KI mice have been generated by inserting the complete human *TARDBP* gene from a BAC, including introns and regulatory elements, into the *Rosa26* locus. These mice show low levels of human TDP-43 expression compared with their endogenous TDP-43, absence of inclusions or gliosis, and a mild age-dependent motor dysfunction, which may give insight into early-stage disease (Gordon et al., 2019).

#### Genomic humanisation

Gene targeting, by homologous recombination or CRISPR/Cas9, enables us to make complex changes in mouse genes, including knocking in human genomic loci that carry important sequences for understanding disease and using these to completely replace the endogenous mouse genes. The FUSDelta14 KI heterozygous mice, expressing a partially humanised mutant *FUS* gene, carrying a splice acceptor site mutation that results in a frameshift that causes an aggressive form of ALS in humans, show progressive spinal MN loss, cytoplasmic mislocalisation of FUS and impaired lipid metabolism (Devoy et al., 2017) (Table 1C). An interesting avenue yet to be explored is the full humanisation of ALS genes. The biochemistry of human proteins, such as SOD1, is sometimes different from that of mouse orthologues, which could be relevant for disease modelling (Prudencio et al., 2009; Karch and Borchelt, 2010; Seetharaman et al., 2010). However, full humanisation of a gene in the context of the mouse genome remains technically challenging and may lead to artefactual results arising from altering the mouse cellular pathways. Thus, each model will have to be assessed carefully on a case-by-case basis and with wild-type human gene controls.

#### Generic gene-targeted mutant mouse features for ALS research

Gene-targeted models express the gene of interest at physiological levels and more closely recapitulate the human ALS-causing mutations at both the genetic and biochemical levels. Together with transgenic models, they can also advance our understanding of disease pathomechanisms, as the technology allows the development of inducible or conditional models that dissect the timing and cell specificity of disease processes. For example, FUSΔNLS mice express a truncated FUS protein lacking the nuclear localisation signal (NLS), with floxed exons 13 and 14 followed by stop codons and a polyadenylation signal, allowing Cre-mediated reversal of the MN loss phenotype, giving new insight into the potential effects of ALS therapies at different disease stages (Scekic-Zahirovic et al., 2016) (Table 1C). However, although few ALS KI models have been produced so far, as is clear from Table 1, the phenotypes of KI mice are often mild and progress slowly. For example, KI models of mutant *Vapb* and *Ubqln2* do not show strong, overt ALS features (Table 1D,F). Nevertheless, they are likely essential for understanding disease onset and the very earliest pathogenic mechanisms, and for developing important biomarkers.

## Random chemical mutagenesis

Random mutagenesis of the mouse genome can give unexpected insight into human biology. Although other methods exist, such as the use of viral integration or radiation treatment, the majority of such mutant mouse models described in the literature come from the use of the powerful chemical mutagen ENU (Acevedo-Arozena et al., 2008). Typically, male mice are injected with ENU, left for several weeks until they start to produce mutant sperm and then mated to wild-type females. Their progeny, carrying point mutations, are assessed for phenotype in a forward genetics screen using wide-ranging tests, so that researchers interested in, for example, progressive locomotor mutants, may determine the causative point mutation and explore the underlying mechanism (Potter et al., 2016). This experimental design is also known as a ‘phenotype screen’. In parallel, sperm and DNA from male progeny are banked for ‘genotype screens’, in which researchers assay the DNA (usually tens of thousands of samples from a single large ENU program) for point mutations in their gene of interest. The corresponding stored sperm samples are then used for *in vitro* fertilisation to (re)derive the relevant mouse line (Stottmann and Beier, 2014).

An informative example of an ENU mutant for ALS research was identified in a genotype screen of *Sod1* within a DNA bank at the mouse facility at MRC Harwell in the UK (Table 1A). This strain, on a C57BL/6J background, carries a *Sod1^D83G^* mutation that is orthologous to the human *SOD1^D83G^* fALS mutation. Heterozygous animals only start to show mild locomotor effects at ∼88 weeks of age, but homozygous mutant mice have a striking phenotype that has allowed the separation of *Sod1* LOF acting in the periphery and GOF effects in MN soma arising from mutant mouse Sod1 (Joyce et al., 2015).

### Generic ENU mutant mouse features for ALS research

Depending on the dose of ENU, the progeny will have multiple mutations within their genome, not just in the gene of interest. Thus, these animals must be backcrossed for several generations to segregate away other mutations, and the flanking congenic region must be carefully assessed to avoid any confounding effects of nearby point mutations (Keays et al., 2007).

One of the advantages of working with ENU mice is the possibility of unexpected insights from novel mutations. However, a disadvantage is that a mutation identified in a genotype screen may not guarantee an aberrant phenotype. For example, our own group identified mouse samples with ENU-induced point mutations in *Tardbp*, but extensive assessment of a rederived strain with a truncated TDP-43 (carrying a *Tardbp^Q101X^* mutation) revealed only limited phenotypes (Ricketts et al., 2014). Nevertheless, as our knowledge of the biochemistry and structure of ALS proteins improves, we can parse ENU mutants by protein domain to help decide which mice to rederive for study. Thus, in follow-up work, our group went on to investigate two more *Tardbp* mutants, one carrying a mutation (M323K) within the low complexity domain (LCD), and the other with a mutation (F210I) in the second RNA-binding domain (RRM2) of TDP-43. The LCD mutation results in a progressive loss of spinal MNs, whereas the RRM2 mutation behaves as a LOF. Importantly for ALS research, transcriptomic analysis of these mice showed that C-terminal TDP-43 mutations lead to a TDP-43 gain-of-splicing function when mutations are expressed at physiological levels. This mutant TDP-43 GOF affects the splicing of a subset of genes not previously known to be controlled by TDP-43, leading to the appearance of new exon exclusion events called ‘skiptic exons’ that are, at least partially, also present in human TDP-43-ALS fibroblast cells (Fratta et al., 2018) (Table 1b).

The TDP-43 ENU mutants highlight another property of ENU mutagenesis: using this method it is relatively straightforward to generate allelic series of animals that may help us dissect protein function.

## Transgenic compared with gene-targeted and ENU mouse models for ALS research

Mice and humans are different animals, and we should not expect a single mouse model to fully recapitulate the entire trajectory of human neurodegenerative disease. We have summarised the key features of two generic types of mouse model – transgenic animals and those that express the gene/mutation of interest at physiological levels – looking at the ease of making each model, and at the types of phenotypes they develop, which usually vary largely because of different expression levels of the gene of interest.

For ALS alleles with dominant modes of inheritance, or where researchers wish to alter protein structure to help dissect function, we have a choice of the type of model to study, and each generic model has different applications (Table 1). Physiological models (gene-targeted and ENU models) have slower progression of phenotypes, which are less severe than in transgenic mice, presumably because proteins are not being overexpressed. However, they maintain correct spatial and temporal levels of expression, which is crucial to avoid the dose-dependent toxicity of some proteins, allowing the study of interactions between the gene/protein of interest and its partners within a pathway over the animal's life span. Moreover, the late disease onset in these mice is useful for gaining insight into the pre-symptomatic stages, and to identify early biomarkers. However, slow phenotypes make these animals expensive to study, as statistically significant differences from their wild-type littermates may only arise after a year or more of life, incurring significant husbandry costs. Nevertheless, they provide a wide window onto early-stage disease processes. In contrast, transgenic animals tend to express strong phenotypes and clear MN loss, making them potentially more relevant to end-stage processes. These differences are exemplified by *Vapb* and *Ubqln2* KI models that have been valuable to study potentially impaired autophagy and proteasomal degradation mechanisms in ALS pathogenesis, but which do not develop the motor impairment or MN loss that can arise in their transgenic counterparts (Table 1D,E).

Mice carrying the TDP-43 Q331K fALS mutation (Table 1B) offer another comparison between gene-targeted KI and transgenic strains. *Tardbp^Q331K^* KIs model the toxic TDP-43 GOF, providing insight into the splicing alteration and the protein autoregulation impairment while reproducing aspects of frontotemporal dementia, although they do not show TDP-43 inclusions or MN loss (White et al., 2018; Fratta et al., 2018). In contrast, the TDP-43 Q331K transgenic line, with transgene expression driven by the mouse prion promotor, shows MN loss, muscle degeneration and neuromuscular junction loss with motor impairments, but only when the transgene is overexpressed above a threshold, confirming the dose-dependent toxic effects of TDP-43 expression (Arnold et al., 2013). Other conditional transgenic lines, such as hTDP-43ΔNLS mice devoid of the TDP-43 NLS, model the toxicity caused by cytoplasmic accumulation and nuclear depletion of TDP-43 (Igaz et al., 2011) (Table 1B).

In summary, several different types of mouse model have been developed worldwide with the aim of reproducing ALS-like phenotypes for functional dissection (Table 1), and it is clear that having access to both transgenic and endogenous mice for each ALS gene could help build a comprehensive picture of the effects of different human ALS mutations.

## Making use of all available ALS-related mouse strains

ALS is probably not a single disease, but arises sporadically and from mutations in a number of genes with varied functions. Mouse models can help us to unravel this complex picture by comparing phenotypes across ALS gene models (Fig. 1). For example, rNLS8 mice, a transgenic model expressing cytoplasmic TDP-43, showed that reactive microglia have an important role in rescuing MN degeneration caused by cytoplasmic TDP-43 expression (Spiller et al., 2018). This is in contrast to *SOD1^G93A^* transgenics, in which transplant of wild-type microglia significantly delays MN degeneration (Wu et al., 2006; Beers et al., 2006). These contrasting results highlight that there are specific disease trajectories in different mouse models, which is also likely the case in ALS patients. Therefore, moving forward, it will be critical to use a variety of models (mouse and other) to understand ALS pathogenesis more broadly, and there remains a need for more models for different ALS genes.

## What is a good mouse model?

This brings us to a key question: what is a good mouse model? The short answer is the animal that is most informative for the underlying biology/disease under study. However, a more considered answer is that researchers must first define the features they are interested in, then chose the most appropriate model. Or better, choose different models to study the disease as broadly as possible. For example, are we looking for subtle, early molecular changes in spinal MNs, or are we interested in models with upper and lower MN death, or are we focusing on the role of glia?

There are many other factors to obtaining a useful mouse model and here we have discussed just one aspect – albeit the crucial one – of model design, i.e. how was the model generated. However, a mouse model's phenotype also depends on the same factors as in humans, including sex and genetic background, which can have profound effects on how a mutation manifests. This is why we have been careful to include these descriptors in Table 1 (Bruijn et al., 2004; Heiman-Patterson et al., 2011; Mancuso et al., 2012; Nardo et al., 2013). Similarly, the environment can markedly affect disease manifestation; for example, environmental enrichment (such as running wheels and nesting material) can increase the life span and behavioural performance in SOD1-G93A mice (Sorrells et al., 2009). Conversely, single housing is a cause of stress in mice and can lead to decreased life span (Kalliokoski et al., 2014), whereas good physical and social interactions positively affect animal welfare (Sundberg and Schofield, 2018).

Mouse models remain necessary for studying ALS, which is a collection of diseases that are not – as far as we know – cell autonomous and that involve different systems, including the immune system. Animal models provide a complex *in vivo* environment of tissues and cell–cell interactions, which are fundamental for the study of complex neurodegenerative diseases, such as ALS, in which the interactions between glia, MNs and muscle are likely necessary for disease development.

## The final word

In gathering the information for this Review to populate the comprehensive Table 1, we found many inconsistencies in the literature describing new mouse lines. It is crucial that descriptions are as complete as possible to define the specific pathology of a model, including reporting the absence of important features, such as MN degeneration, and other negative results. The use of Animal Research: Reporting of *In Vivo* Experiments (ARRIVE) guidelines (Kilkenny et al., 2014) will improve reporting of mouse model phenotypes. In addition, it is critical to make new models freely available via The European Mouse Mutant Archive (EMMA) or The Jackson Laboratory (JAX).

The complexities of ALS are clearly exemplified by the wide array of phenotypes arising in the plethora of mouse models available. If anything, the past decades of research into ALS have shown us that to improve our understanding of disease pathogenesis, the community must embrace its complexities and work with different models. In the near future, integrating data from multiple sources, mouse and human, *in vivo* and *in vitro*, should allow us to build a more complete picture of health and disease states, over a lifetime. However, ultimately, we must relate our findings back to humans, and cell, organoid and clinical models remain essential for cross-referencing and validating the findings from mouse studies.
